# The Quebec Pregnancy Cohort – Prevalence of Medication Use during Gestation and Pregnancy Outcomes

**DOI:** 10.1371/journal.pone.0093870

**Published:** 2014-04-04

**Authors:** Anick Bérard, Odile Sheehy

**Affiliations:** 1 Faculty of Pharmacy, University of Montreal, Montreal, Quebec, Canada; 2 Research Center, CHU Ste-Justine, Montreal, Quebec, Canada; Queen's University, Canada

## Abstract

**Purpose:**

We evaluated the potential and the validity of the Quebec Pregnancy Cohort (QPC) as a research tool in perinatal pharmacoepidemiology.

**Methods:**

The QPC was built by linking four administrative databases: RAMQ (medical and pharmaceutical data), Med-Echo (hospitalizations), ISQ (births/deaths), and MELS (Ministry of Education data). A self-administered questionnaire was sent to a random sample of women to collect lifestyle information. The QPC includes data on all pregnancies of women covered by the Quebec provincial prescription drug insurance between 1998 and 2008. Date of entry in the QPC is the first day of pregnancy, and women are followed during and after pregnancy; children are followed after birth up until 2009. The prevalence of prescribed medications before, during and after pregnancy was compared between time-window. Pregnancy outcomes were also estimated among pregnancies ending with a live born infant.

**Results:**

The QPC included 289,688 pregnancies of 186,165 women. Among them, 167,398 ended with a delivery representing 19.4% of all deliveries occurring in the Province of Quebec between 1998–2009. The total frequency of abortions was 35.9% in the QPC comparable to the 36.4% observed in the Province of Quebec. The prevalence of prescribed medication use was 74.6%, 59.0%, and 79.6% before, during and after pregnancy, respectively. Although there was a statistically significant decrease in the proportion of use once the pregnancy was diagnosed (p<.01), post-pregnancy prescribed medication use returned above the pre-pregnancy level. The prevalence of pregnancy outcomes found in the QPC wer***e*** similar to those observed in the Province of Quebec.

**Conclusion:**

The QPC is an excellent tool for the study of the risk and benefit of drug use during the perinatal period. This cohort has the advantage of including a validated date of beginning of pregnancy giving the possibility of assigning the exact gestational age at the time of maternal exposure.

## Introduction

Since the thalidomide disaster of the 1960s, there has been an increased general awareness of the potential side effects of drug exposure during pregnancy.[1] The resulting effect is that physicians are now very cautious about prescribing medications during pregnancy.[2] At least half the pregnancies in North America are unplanned,[3] resulting in millions of women and unborn infants exposed to prescribed medications during the organogenesis period because women did not know they were pregnant. Because the Food and Drug Administration (FDA) and Health Canada do not permit the inclusion of pregnant women in clinical trials assessing drug efficacy, data on the safety of drug exposure during pregnancy before the medication is on the market are scarce. Since, from an ethical point of view, it is almost impossible to randomize pregnant women to receive prescribed medications not known to be safe for the foetus, the collection and follow-up of observational data is the only ethical way to close the knowledge gap between the limited value of animal studies and human pregnancy exposures.

To date, the majority of studies on the risks and benefits of medication use during pregnancy include small sample sizes, lack of statistical power, or have sub-optimal study designs to investigate rare outcomes such as congenital malformations, low-birth-weight (LBW) or prematurity.[4], [5] Furthermore, although pregnancy outcomes immediately after birth are studied, few data exist on the long-term neurobehavioral development of children exposed to prescribed medications in-utero.[6]–[8] To circumvent these limitations, in recent years, large national administrative databases or registries have been increasingly used in the field of perinatal pharmacoepidemiology.[9]–[13] Not surprising, this produced contradictory results between large database studies and small field studies.[6], [14]

Given that access and delivery of health care vary from country to country, and that large administrative databases may have missing data on important potential confounders such as smoking, caffeine and folic acid intake, and alcohol use, the Quebec Pregnancy Cohort (QPC) was established to study short- and long-term effects of medication use during gestation on the mother and child as well as the neurodevelopment of school aged children. The QPC also provides the opportunity to study other important perinatal risk factors given that a substantial number of pregnant women do not take prescribed medications during gestation. With this paper, we aim to present the QPC and provide information on prevalence of prescribed drugs during the perinatal period as well as baseline population-based results in order to highlight the registry's potential for perinatal pharmacoepidemiologic research. We hypothesized that the QPC would provide accurate and valid information on prescription drug consumption, pregnancy outcomes and prevalence of chronic diseases during the perinatal period.

## Methods

### Ethics Statement

The linkages between administrative databases and the self-administered questionnaire were approved by the Ethics Committee of Ste-Justine's Hospital. The Commission d'accès à l'information (CAI) of Quebec gave the authorization for the acquisition of the data necessary for the creation of the QPC. All women who responded to the questionnaire provided informed consent.

### The Quebec Pregnancy Cohort

The QPC is an ongoing population-based cohort with prospective data collection built with the linkage of four administrative databases from the province of Quebec, Canada. For each individual, data in the Régie de l'Assurance Maladie du Québec (RAMQ), Med-Echo, the birth and death registries of l'Institut de la Statistique du Québec (ISQ) and the Ministère de l'éducation, des loisirs et des sports du Québec (MELS) are linked by a unique encrypted identifier. The QPC currently contains data on all pregnancies that occurred between January 1997 and September 2009 and were covered by Quebec's Public Prescription Drug Insurance Plan for at least 12 months before the first day of gestation and during pregnancy. Data on the mothers and children after the end of pregnancy are also collected. An update of the QPC is currently underway to include medical, pharmaceutical, and hospital data on new pregnancies, as well as follow-up data from 2010–2013 on mothers and children for pregnancies that are already present in the QPC.

The RAMQ provides medical coverage to all Quebec residents and pharmaceutical coverage to 43% of the overall Quebec population (welfare recipients, employees who do not have medication coverage from their employer or spouse's employer, and individuals 65 years of age or older). The RAMQ database in the QPC represents 36% of women between 15–45 years of age[15] and the RAMQ Demographic file includes information on age, sex, postal code, date of death, and dates of coverage by drug plan (welfare recipients, employees not covered otherwise, and individual ≥65 yr of age). The RAMQ Medical Services file contains detailed information on all medical services, including physician-based diagnosis and therapeutic procedures, diagnoses coded according to the International Classification of Diseases, ninth and tenth revisions (ICD-9, ICD-10)[16], [17], as well as the date and the type of institutions where the medical procedures were performed. Health care provider characteristics are also included. The RAMQ Prescription Drug file covers information on all filled prescribed medications, the prescribing physician and dispensing pharmacist, drug name, dosage, formulation, quantity dispensed, date and duration of the dispensation for publicly insured people. Data in the RAMQ Prescription Drug file have been validated and found to be highly reliable.[18] Furthermore, pregnant women insured by the RAMQ prescription drug insurance plan have been shown to be of lower socioeconomic status but comparable to those insured by private insurance companies in terms of their comorbidity profiles, use of prescribed and non-prescribed medications use, and their health services utilization such as physician visits and hospitalizations.[15]

The Med-Echo database records all acute care hospitalization in the province of Quebec, including the length of gestation (defined from the first day of the last menstrual period to the end of pregnancy validated by ultrasound) and birth weight. Med-Echo is the first administrative database to give exact gestational age at the end of pregnancy, which is a great advantage for studies on drug use during gestation where timing of exposure is essential. Data on physician-based medical diagnoses found in Med-Echo have been validated.[19] ISQ provides demographic information on the mother, father, and baby as well as birth weight and gestational age for live births and stillbirths. Data recorded in the ISQ database have been compared to medical charts and found to be complete and valid.[20] The MELS database gives information on use of specialized services at the elementary school level such as speech therapist or psycho-educator.

In order to create the QPC, the linkage between the databases was performed using a patient unique encrypted identifier (RAMQ and Med-Echo), and mothers' and babies' dates of birth, first names, and family names (RAMQ, ISQ, and MELS). Each subject's unique encrypted identifier is provided to the research team by the RAMQ. Pregnant women are identified by a prenatal visit in the RAMQ database or by a therapeutic procedure related to pregnancy in RAMQ or Med-Echo (e.g., ultrasound, amniocentesis, procedures related to a planned or spontaneous abortion, delivery, etc.). In the QPC, women are followed from the beginning of pregnancy, defined as the first day of the last menstrual period confirmed by ultrasound, until the end of pregnancy (planned or spontaneous abortion, or delivery, whichever comes first). The status of the newborn (stillbirth or livebirth) is obtained via the ISQ database. Women are treated and followed prospectively as part of the usual health care management during and after pregnancy, and children are similarly followed after birth. Data on physician-based diagnoses of major congenital malformations in the RAMQ and Med-Echo databases have been found to be valid.[21], [22]

### Self-administered questionnaire variables

In order to collect information not present in the administrative databases, 8,505 pregnancies were randomly selected among pregnancies ending with a live born between January 1998 and December 2003. A self-administered questionnaire was mailed to them to collect informations on lifestyle variables, socio-demographic information, weight and height at the beginning of the pregnancy, weight gain during pregnancy, natural health product use, folic acid intake, and data regarding pregnancy history. In order to maximise our response rate the questionnaires were sent twice and a toll-free telephone line was provided to aid women who required further information. A monetary incentive ($5.00CAN) was also sent for each returned questionnaire. Information collected with the self-administered questionnaire was linked to the QPC using the patient unique encrypted identifier numbers.

### Baseline characteristics and prevalence of prescribed medication use during the perinatal period

Baseline data on the QPC are presented here for the study period presently available (1997–2009). Characteristics of the women were assessed on the first day of gestation (1DG); defined as the first day of the last menstrual period confirmed by ultrasound available in the MED-ECHO and ISQ databases. Prescribed medication exposure included all drugs covered by the RAMQ obtained on prescription and dispensed by a pharmacist. The drugs covered over 7,000 drugs listed on the “List of Medications”, published periodically by the RAMQ.[23] Prevalences of prescribed medication exposure are presented according to the 3 following study intervals: 1) before pregnancy (12 months before the 1DG), 2) during pregnancy (1DG until the end of pregnancy (miscarriage, abortion or delivery)), and after pregnancy (12 months after the end of pregnancy). The pregnancy was also divided by trimesters. The 1^st^ trimester was defined as the time from the 1DG until the 14^th^ completed week of gestation, the 2^nd^ trimester (between the15th week and the 25^th^ completed week of gestation), and the 3^rd^ trimester (between the 26^th^ week until the end of the pregnancy). Exposure to prescribed medication was defined as having at least one prescription filled during the study interval of interest or one prescription filled before the beginning of the interval but with duration overlapping the interval. The prevalence of exposure of the following classes of prescribed medications were also estimated: oral contraceptives (OCs), vitamins, asthma drugs, antidepressants, benzodiazepines, non-steroidal anti-inflammatory drugs (NSAIDs), morning-after pill, synthroid and anti-emetics.

### Pregnancy outcomes

Pregnancy outcomes were evaluated at the end of the pregnancy. Only clinically apparent or detected spontaneous and planned abortions are identified and reported here. Stillbirths were identified in the ISQ database without specific causes; and the prevalence of multiplicity is also presented. Among singleton pregnancies ending with a delivery, prematurity was defined as being born before the 37^th^ week of gestation, and LBW as newborns with a birth weight of less than 2500 g. Infants with major congenital malformations (MCM) were identified among singletons using validated diagnoses of MCM at birth or during the first 12 months of life ((ICD-9 codes: 740–759) excluding (743.6, 744.1, 744.2–744.4, 744.8, 744.9, 747.0, 747.5, 750.0, 752.4, 752.5, 754.6, 755.0, 755.1, 757.2–757.6, 757.8, 757.9, 758.4) or ((ICD-10 codes: Q00–Q99) excluding (Q10, Q162, Q17–Q182, Q184–Q189, Q250, Q270, Q381, Q515, Q516, Q520–Q527, Q53, Q664–Q666, Q69, Q70, Q81–Q84, Q950–Q952, Q954, Q955, Q959) recorded in the RAMQ and MED-ECHO databases.

### Chronic diseases and postpartum depression

The prevalence of the following chronic diseases were considered, and measured in the year before and during pregnancy: diabetes (ICD-9 codes 250–259, 271.4 and 790.2 or ICD-10 codes E10-E14 and R730, or at least one prescription for medications for diabetes, AHFS codes 68∶20.08, 68∶20.20, and 68∶20.92); hypertension (ICD-9 codes 640–642 or ICD-10 codes I10-I15, O10-O16, or at least one prescription for antihypertensive drugs); and depression (ICD-9 codes 296, 309, 311 or ICD-10 codes or at least one prescription for antidepressants). Post-partum depression was defined as having a diagnosis of post-partum depression or depression (ICD-9 codes: 648.4, 296, 300, 309,311 or ICD-10 codes:O906, F300–F302, F308–FF320, F322, F323, F328–F334, F338, F339, F341, F348, F349, F380, F381, F388, F390–F402, F408–F413, F418–F422, F428, F429, F431, F432, F438, F440–F452, F480, F481, F488, F489, F530, F680, F930, F99) in the 2 months after delivery, and in the 12 months after giving birth, separately, considering that post-partum depression can well be detected after the traditional 2 months post-delivery. Diagnostic codes for MCM have been compared using medical chart reviews and found to be accurate and valid.[22]

### Statistical analyses

Characteristics of the women and pregnancy outcomes are presented as proportions for categorical variables and means with standard deviations (SD) for continuous variables. Prevalences of exposure to prescribed medications are presented as proportions of pregnancies exposed for all prescribed medications combined and by class for each study interval. Prevalences of prescribed medications are compared between intervals using McNemar's test. Prevalences of pregnancy outcomes are also presented as proportions. Variables obtained from the self-administered questionnaire were presented as proportion for categorical variables and means with SD for continuous variables. All analyses were conducted using the SAS System for Windows Version 9.1.3 (SAS Institute Inc., North Carolina, USA).

## Results

### Descriptive data and prescribed medications use in the Quebec Pregnancy Cohort

**Figure 1:**
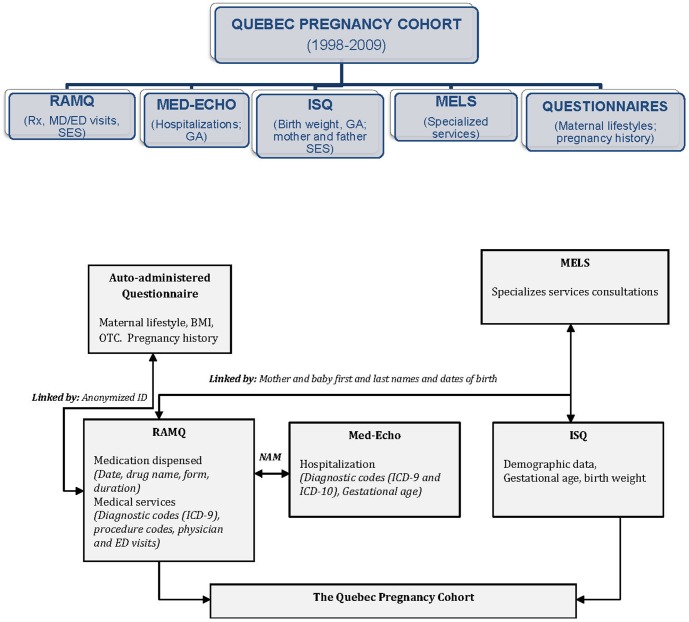
The Quebec Pregnancy Cohort: linkage between the administrative databases used and the auto-administered questionnaire. Abbreviations: Rx, prescription filled; MD/ED, medical and emergency department; SES, social economic status; GA, gestational age; BMI, body mass index; OTC, over-the-counter; ICD-9 and ICD-10, International Classification of Diseases, 9^th^ and 10^th^ revision; NAM, Numero d'assurance maladie (unique personal identification number).

**Figure 2:**
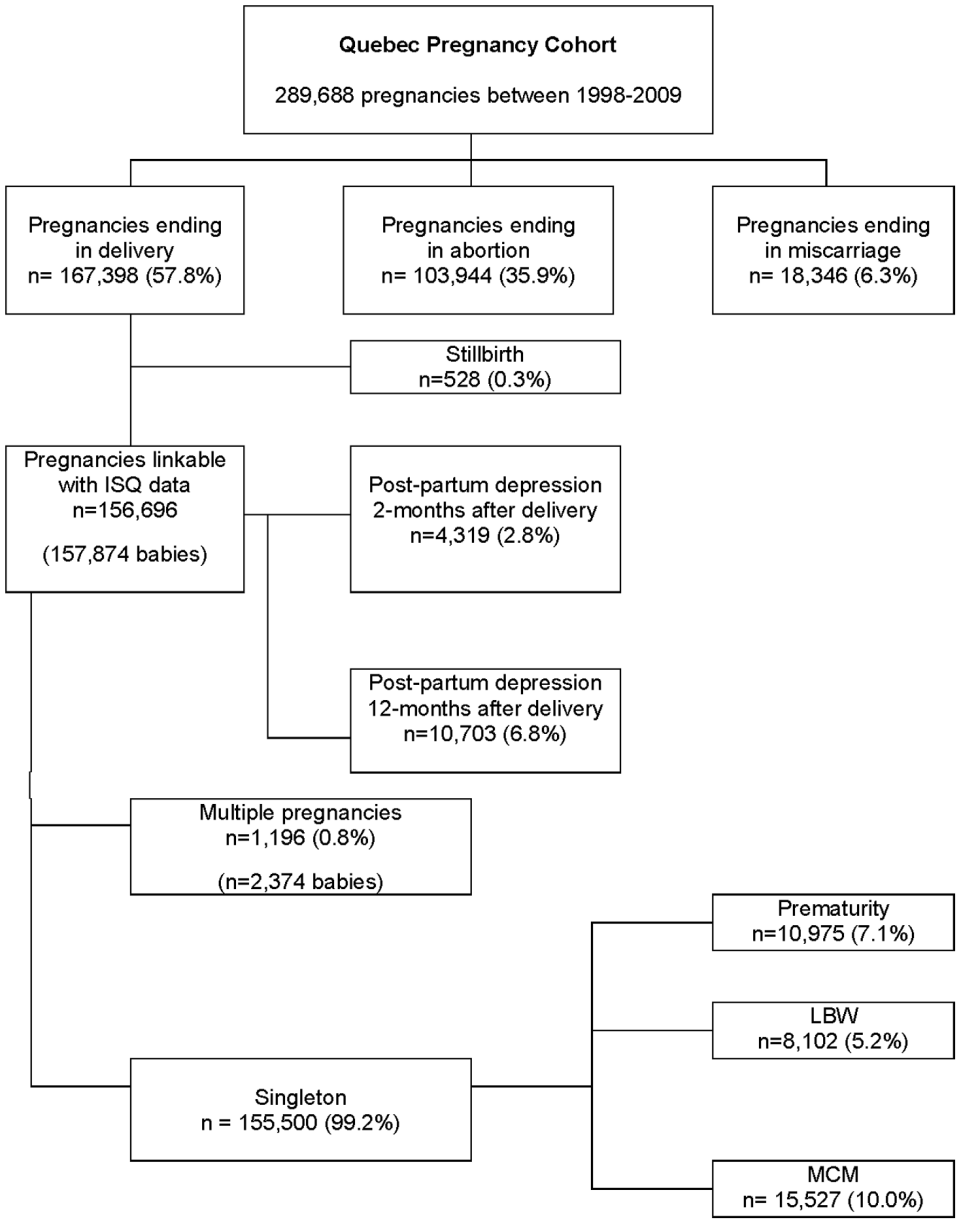
Quebec Pregnancy Cohort and outcomes. Prevalence of pregnancy outcomes during the period 1998–2009.

Figure 1 summarizes the construction of the QPC. For the study period 1998–2008, the QPC was comprised of 289,688 pregnancies and 186,165 women with complete data for the three study intervals of 12 months before pregnancy, during pregnancy, and 12 months after pregnancy. The number of pregnancies per women during the study period ranged from 1 to 13 with a median of 1 pregnancy per women. Among them, 167,398 (57.8%) ended with a delivery, 103,944 (35.9%) with a planned abortion, and 18,346 (6.3%) with a miscarriage (Figure 2). The mean maternal age was 27.8±5.6, and was similar regardless of the pregnancy termination status. Pregnancies of women living in rural area were more likely to end with a delivery than a planned abortion. The number of pregnancies ending with a planned abortion was higher among welfare recipients compared to adherents (workers). Table 1 presents the prevalence of chronic/gestational diabetes, chronic/gestational hypertension, and depression. The overall prevalence of chronic/gestational diabetes was 3.4% in the QPC and the prevalence of essential/gestational hypertension was 6.3%. The prevalence of essential/gestational hypertension was almost 3 times higher among pregnancies ending with a delivery than for those ending with a planned abortion. The prevalence of depression was estimated at 18.4% with a higher prevalence among pregnancies ending with a miscarriage as compared to pregnancies ending with delivery or planned abortion.

**Table 1:** Characteristics of pregnancies included in the QPC.

	All pregnancies	Deliveries	Planned abortions	Miscarriages
Characteristics	n = 289,688	n = 167,398	n = 103,944	n = 18,346
Maternal age (years)– mean ± SD	27.8±5.6	27.8±5.6	27.2±6.5	28.8±6.4
Duration of pregnancy (weeks) – mean ± SD	n.a.	38.7±2.2	14.3±2.3	18.1±4.6
Living in rural area – n (%)	44,726 (15.4)	29,540 (17.7)	12,213 (11.8)	2,973 (16.2)
Welfare recipient – n (%)	81,933 (28.3)	42,602 (25.5)	33,943 (32.7)	5,388 (29.4)
Comorbidities in the year prior and during pregnancy:				
Diabetes (chronic/gestational) – n (%)	9,875 (3.4)	8,374 (5.0)	1,098 (1.1)	403 (2.2)
Hypertension (essential/gestational) – n (%)	18,092 (6.3)	14,399 (8.6)	2,964 (2.9)	729 (4.0)
Depression– n (%)	53,368 (18.4)	28,575 (17.1)	20,638 (19.9)	4,155 (22.7)

n.a =  not applicable.

**Figure 3:**
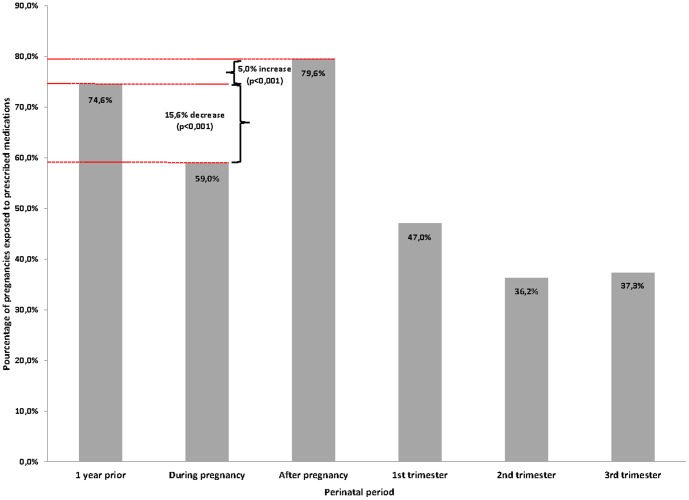
All prescribed medications combined (including vitamins) during the perinatal period.

**Figure 4:**
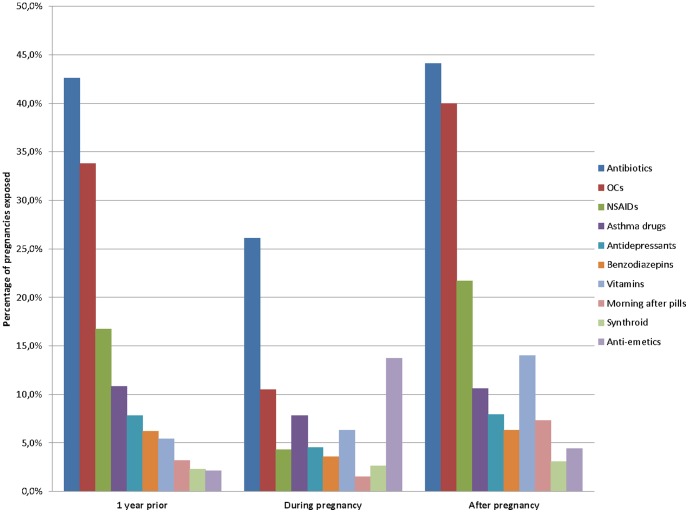
Prescribed medication use by class during pregnancy.

The prevalence of prescribed medication use, including vitamins, in the year before pregnancy was 74.6%; 59.0% during pregnancy (47.0% in the 1^st^ trimester, 36.2% in the 2^nd^ trimester, and 37.3 in the 3^rd^ trimester); and 79.6% in the year after pregnancy (Figure 3). Although there was a statistically significant decrease in the prevalence of prescribed medications use once the pregnancy was diagnosed (p<.001), prescribed medication use was highest in the post-pregnancy period (p<.001). During pregnancy, prescribed medication users were of similar age as non-users (27.5 years (standard-deviation (SD) 6.0) vs. 27.9 years (SD 5.9)). The most frequently prescribed medications used in the year prior to pregnancy were antibiotics (42.6%), OCs (33.8%), NSAIDs (16.7%), asthma drugs (10.8%), and antidepressants (7.8%) (Figure 4). During pregnancy, the most frequently prescribed medications used were antibiotics (26.1%), anti-emetics (13.7%), OCs (10.5%), asthma drugs (7.8%), vitamins (6.3%), and antidepressants (4.5%); in the year after pregnancy, these were antibiotics (44.1%), OCs (40.0%), NSAIDs (21.7), vitamins (14.0%), asthma drugs (10.6%), and antidepressants (7.9%). The exposure to antibiotics during pregnancy decreased from 16.3% during the 1^st^ trimester to 11.4% and 11.6% during the 2^nd^ and 3^rd^ trimester, respectively. The prevalence of anti-emetics drugs exposure during the 1^st^ trimester was 12.0% and decreased during the 2^nd^ and the 3^rd^ trimesters. Antidepressants exposure decreased over the course of pregnancy (4.3%, 2.4%, and 1.6% for the 1^st^, 2^nd^ and 3^rd^ trimester, respectively).

### Baseline data on pregnancy outcomes in the Quebec Pregnancy Cohort

The prevalence of stillbirths in the QPC was 3.2 per 1000 pregnancies as compared to 4.2 in the Province of Quebec between 2006-2010. Amongst women who had a delivery, it was possible to find at least one linkable baby in the ISQ for 156,696 (93.6%) of them. The prevalence of singleton was 99.2% (155,500) and 1,196 multiple pregnancies were observed (1,188 twins, and 8 triplets). Figure 2 shows the prevalence of pregnancy outcomes. The prevalence of prematurity in the QPC was 7.1%, and the prevalence of LBW was 5.2%. The prevalence of MCM in the QPC was estimated at 10%; the annual rate per 1 000 pregnancies between 1998 and 2009 are presented by organ system in Table 2. Malformations of the musculoskeletal system were the most frequent MCM with an annual rate of 38.8/10,000 pregnancies, followed by malformations of the circulatory system. Post-partum depression prevalence at 2-months after delivery was estimated at 2.8%; the prevalence of post-partum depression was 6.8% at 12-months post-partum (Table 1).

**Table 2:** Major Congenital malformations by organ system among 155,500 singleton pregnancies.

		Annual rate
Description	n (%)	per 10,000 pregnancies
Any major congenital malformation	15,527 (10.0)	998.5
Nervous system	904 (0.6)	58.1
Eye, ear, face and neck	895 (0.6)	57.6
Circulatory system	3,560 (2.3)	228.9
Respiratory system	716 (0.5)	46.0
Cleft palate and/or lip	1,195 (0.8)	14.5
Digestive system	225 (0.1)	76.8
Genital organs	1,424 (0.9)	91.6
Urinary system	1,212 (0.8)	77.9
Musculoskeletal system	6,041 (3.9)	388.5
Other	1,869 (1.2)	120.2
Chromosomal abnormalities	544 (0.4)	35.0

### Results on socio-economic status, lifestyles, and pregnancy history from the self-administered questionnaire

The final response rate for the mailed questionnaires was 39.5% (3,273 of the 8,505 randomly selected women). Responders were similar to non-responders regarding maternal age, region of residence (urban vs. rural dwellers), maternal marital status (living alone vs. co-habiting), and RAMQ drug insurance status (welfare status vs. adherents (workers)), gestational age, birth weight, and rate of MCM, healthcare use (rate of hospitalisation, and emergency department (ED) visits, physician visits, number of prenatal visits, visits to obstetricians, and dispensed co-medications during pregnancy), presence of chronic co-morbidities such as hypertension, diabetes, asthma, and depression, prevalence of multiplicity, newborn gender, and calendar year of delivery (data not shown here but are presented in Moussally and Bérard).[24] Table 3 presents the characteristics of responders. The majority of responders were Caucasians living with a partner, working, and had an annual income of less than 30,000CAN$; almost 40% of responders had a post-secondary education. Responders were of normal weight and gained a mean of 16 kg during gestation. Once the pregnancy was diagnosed, there was an uptake of physical activity and multivitamin use, and a decrease of OTC medication exposure, caffeine and alcohol intake, and illicit drug use. Natural health product use remained low during and after pregnancy in this population. Maternal smoking decreased during pregnancy but increased again after the end of gestation (49.7%, 35.0%, and 38.9% for the period before, during, and after pregnancy, respectively). Newborns were exposed to high levels of second hand smoking either by the mother alone (38.9%), father alone (36.4%) or both parents (23.5%). Responders had a prevalence of MCM comparable to the provincial statistics of Quebec (5.6% vs. 6.7% for Quebec).[25] Sixty-one percent (61%) of responders breastfed their infant for a mean duration of 6.5 months (SD 6.8), and 78% of children went to daycare starting at 20.8 months of age on average (SD 13.3).

**Table 3:** Maternal and children characteristics amongst the 3,273 mothers who returned the self-administered questionnaire.

Characteristics	
BMI (kg/m^2^) - (mean ± SD) (n* = 2,761)	23.5±5.2
Weight gain during pregnancy (kg) - (mean ± SD) (n = 2,536)	16.0±5.7
Breastfeed - (%) (n = 3,231)	61.3
Duration of breastfeeding (months) - (mean ± SD) (n = 1,972)	6.5±6.8
Child in day care - (%) (n = 3,240)	77.9
Age at the entry date in day care (months) - (mean ± SD) (n = 2,448)	20.8±13.3
Ethnic groups: - (%) (n = 3,104)	
Caucasian/white	88.5
Black	2.3
Hispanic	1.5
Asian	1.6
First nation	0.5
Other	5.5
Education level: - (%) (n = 3,192)	
Secondary 1&2	8.8
Secondary 3,4 & 5	38.7
College (CEGEP)	23.5
University	15.2
Other	13.9
Living with a partner - (%) (n = 3,215)	82.9
Worker - (%) (n = 3,042)	56.3
Gross annual family income: (CAN$) - (%) (n = 3,191)	
$18 000 or less	34.6
$18 001–$30 000	27.9
$30 001–$46 000	19.7
$46 001–$67 000	11.0
$67 000 and more	6.8
Lifestyles before pregnancy: - (%)	
Physical activities (n = 1,613)	59.6
Multivitamin intake (n = 3,037)	26.9
OTC medications use (n = 3,169)	71.6
Caffeinated beverage use (n = 3,222)	86.7
Alcoholic beverage consumption (n = 2,884)	67.6
Illicit drug use (n = 3,246)	15.9
Lifestyles during pregnancy: - (%)	
Physical activities (n = 1,982)	68.2
Multivitamin intake (n = 2,779)	84.2
OTC medication use (n = 3,186)	44.9
Caffeinated beverage use (n = 3,110)	71.8
Alcoholic beverage consumption (n = 3,104)	19.2
Illicit drug use (n = 3,217)	4.8
Smoking status of the mother: - (%)	
Prior to pregnancy (n = 3,195)	49.7
During pregnancy (n = 3,113)	35.0
Smoking status after the birth of the baby: - (%)	
Mother only (n = 3,160)	38.9
Father only (n = 3,041)	36.4
Both (n = 2,947)	23.5
Natural health product use: - (%)	
During the year before pregnancy (n = 3,270)	16.2
1^st^ trimester (n = 3,269)	9.9
2^nd^ trimester (n = 3,269)	9.5
3^rd^ trimester (n = 3,269)	9.9
During the year after pregnancy (n = 3,269)	14.7
Pregnancy history of: - (%)	
Premature birth (n = 1,992)	14.5
Low birth weight babies (n = 1,989)	10.7
Children with congenital malformations (n = 1,980)	5.6
Spontaneous abortions (n = 3,208)	22.7
Planned abortions for genetic reasons (n = 3,208)	4.6

*Because of missing value, some samples are lower than 3,273.

## Discussion

Baseline statistics from the QPC have highlighted the fact that up to 59% of pregnant women in Quebec take prescribed medications during gestation. The most used medications during pregnancy were antibiotics, anti-emetics, and NSAIDs. Prescribed medication exposure decreased significantly once the pregnancy was diagnosed but increased above pre-pregnancy levels immediately after delivery, partly explained by the uptake of vitamins (2.8% before to 6.3% after the pregnancy) and antibiotics (16.3% before and 26.1% after). Planned abortion, premature birth, and LBW prevalence found in the QPC were similar to those observed in the Province of Quebec for the same time period.

Once the pregnancy was diagnosed, significant lifestyle changes were made such as decreased tobacco, alcohol and illicit drug use, and caffeine intake. Almost 40% of newborns were exposed to second-hand smoking. Finally, 61% of mother's breastfed their infants for 6 months on average, and the majority of children were in daycare at 20 months of age.

Data from the QPC showed that at least 56.7% of pregnant women had an on-going medication prescription during gestation, whether vitamins were considered or not. Although there is inter-country variation in the prevalence of medication exposure during gestation[26], [27] (86% The Netherlands, 96% Germany, 74%–100% France, 68%–100% USA, 46%–100% Finland, 44% Denmark), partly explained by cultural differences, drug reimbursement plans, definitions of drug exposure within studies, and maternal age or other maternal characteristics, it remains that the QPC is comparable to others. Indeed, the prevalence of prescribed medication use during pregnancy found in the QPC is similar to those reported by Lacroix et al.[28] in France where 42% of pregnant women used anti-infective drugs (vs. 44.3% of antibiotics users in the QPC), and 20% were using metoclopramide (vs. 23.2% of anti-emetic users in the QPC).

In the QPC, 35.9% of pregnant women had a planned abortion. This is comparable to the general population of Quebec where the annual rate of planned abortion for the same period was 36.4 per 100 deliveries.[29] However, the planned abortion rate in the QPC is higher than what has been observed in the US where it is 23.3 per 100 pregnancies.[30]_ENREF_20 This could partly be explained by the fact that women have free and direct access to planned abortions in the Province of Quebec. Six percent of pregnant women in the QPC had a clinically apparent spontaneous abortion, which is lower than the 10%–15% reported elsewhere.[31], [32] Categorisation of spontaneous and planned abortions within the QPC is made with different procedure codes, which limits any potential outcome misclassification (over-estimation of spontaneous abortions and under-estimation of planned abortions) that could result from patient or physician reported assessment of outcome in other settings. Within the QPC, prematurity was estimated at 7.1% similar to the Province estimate (7.6%).[33] This is also similar to what has been reported elsewhere in Canada in 2004 for singleton births (8.2%)[34]_ENREF_21 as well as in the United Kingdom (6.5%) and in Belgium (8.4%)[35]. It is however higher than in France where up to 6.5% of births are preterm,[36] and lower than in the US where a prevalence of prematurity of 10.8% has been reported.[37] The prevalence of LBW found in the QPC was 5.2% compared to 5.7% in the Province of Quebec.[33] As for multiplicity, the reported prevalence in the QPC is lower than what has been reported for the whole population of Quebec (0.8% in the QPC vs. 2.9% in Quebec overall).[38] This can partly be explained by the fact that the most important risk factor for multiple births is infertility treatments[37] which are costly and were not reimbursed by the RAMQ until recently.

At least one baby could be linked to the ISQ database for 93.6% of all pregnancies with a pregnancy ending with a delivery. The baseline prevalence of major congenital malformations (MCM) was estimated at 10% during the study period. The rate of MCM in Quebec is known to be higher than the usual reported rate of 3–5%,[39] and can be explain by the founder's effects and cluster region with very high rates of MCM.[40] In the absence of a system for recording and monitoring anomalies in Quebec, there is very little accurate information on the overall incidence of children with birth defects. Annual rate of cleft palate and/or lip observed in our cohort was 14.5/10 000 pregnancies comparable to 15.3 in the same period in the Province of Quebec.[41]

The overall prevalence of chronic/gestational diabetes was 1.1%–5.0% depending on the pregnancy outcome considered. This is comparable to the prevalence of diabetes in women under the age of 40 in the Province of Quebec (2.0%).[42] The prevalence of pre-existing hypertension and gestational hypertension was 1% as compared to 5% in the Province of Quebec.[43] We have found a prevalence of essential/gestational hypertension of 6.3% in the QPC. The overall prevalence of depression in the QPC was 18.4% which is almost identical to the prevalence observed in the Province of Quebec (18.6%).[44]

Finally, 6.8% of pregnant women in the QPC had post-partum depression disorders diagnosed in the year following delivery. This is comparable to studies which reported rates of postnatal depression disorders of 7.3% in the first three months after delivery[45] and 10.4% at 6-months postpartum.[46]

Data in the QPC showed that 61% of mothers breastfed their infants for 6 months on average. Although few other population-based data are available for breastfeeding, this is in agreement with the current recommendations on breastfeeding.[47] The majority of children were in daycare at 20 months of age. It has been shown that children of families of lower socio-economic status who attend day care have better cognitive development comparable to children staying at home.[48] Given the socio-economic status of women in the QPC, day-care attendance is expected to result in better outcome for these children. Furthermore, the publicly funded day care program in Quebec makes it affordable for families to place a child in daycare.

Although there has been an increase in the assembly of cohorts of pregnant women over the past years, the QPC offers an interesting range of variables and is one of the few that gives exact validated gestational age, which is essential in perinatal pharmacoepidemiologic studies. The QPC is population-based among women insured by the RAMQ prescription drug insurance plan for their prescribed medications, and includes physician-based prospective diagnoses and procedure codes, data on prescription fillings including date of filling, duration of prescription and dosage, and has validated diagnoses of MCM. The majority of prescribed medications are reimbursed by the RAMQ prescription drug insurance plan including prescribed OTC. However, the non-prescribed OTC medicines are not included in the RAMQ database. The prevalence of OTC medicines used before and during pregnancy obtained from the self-administered questionnaire was high, 71.6% and 44.9% for before and during pregnancy, respectively. In fact, there was no difference in the distribution of vitamin consumption before and during pregnancy when we compare pregnant women insured by the RAMQ-Rx and those insured by private drug insurance programs, regardless of their work status.[15]

Given the prospective nature of the data collected on prescription fillings, information on medication use do not suffer from recall bias, and appropriate medication filling algorithms can limit bias resulting from drug non-compliance. Filled prescribed medications have been compared to self-reported data on mediation use during pregnancy, and have been found to be valid for all medication classes.[49]

The QPC is one of the few cohorts, to our knowledge, that is documenting cognitive development of children (in the form of special services use), and provides long-term follow-up of mothers and children. Given the administrative nature of the databases used, data on smoking, alcohol and illicit drug use as well as caffeine and folic acid intake, and maternal weight and weight gain during pregnancy are missing. Although this is a limitation, it can be circumvented by using appropriate study designs and medication filling algorithms. In addition, an attempt has been made to quantify the bias that would result from the absence of this information on study results;[50] it has also been shown that pregnant women having medication insurance from the RAMQ prescription drug insurance plan had similar characteristics and medical history than those who had private drug insurance plans.[15]

The QPC has previously been used to assess risks and benefits of drug use during pregnancy.[9], [10], [14], [51] Although studies on the risks and benefits of medication exposure during pregnancy only include women covered by the RAMQ prescription drug insurance plan (36% of women between 15–45 years of age), Bérard and Lacasse[15] have shown that socio-economic status is an effect modifier, and thus does not affect internal validity of etiologic studies but might affect generalizability. Furthermore, studies on risk factors other than prescribed medications are not vulnerable to this limitation because access to health care is universal in the Province of Quebec.

Finally, we have shown that baseline statistics from the QPC were comparable, for the most part, to similar statistics from other pregnancy cohorts or populations published elsewhere. Baseline results presented here mostly highlighted the fact that a high prevalence of pregnant women take prescribed medications during gestation and that more research needs to be made in this special population to fully assess and quantify the risks and benefits of medication exposure for the mother and child. The QPC is a pregnancy, mother and child cohort that has the potential to fill this knowledge gap.

### Conclusion

In conclusion, the QPC turns out to be an excellent tool to measure the benefits and the risks of using medications during the perinatal period. The large number of pregnancies in the cohort provides the power needed to measure rare pregnancy outcomes. The QPC provides information to measure potential confounding variables, especially exact gestational age at the end of pregnancy validated by ultrasound, which ensures accurate timing of drug exposure.
